# Microplastic Contamination in Soils: A Review from Geotechnical Engineering View

**DOI:** 10.3390/polym13234129

**Published:** 2021-11-26

**Authors:** Mehmet Murat Monkul, Hakkı O. Özhan

**Affiliations:** Department of Civil Engineering, Yeditepe University, İstanbul 34755, Turkey; hakki.ozhan@yeditepe.edu.tr

**Keywords:** microplastics, soil, polymers, geotechnics, landfills, geosynthetics, GCL, clay liner, hydraulic conductivity, plastics

## Abstract

Microplastic contamination is a growing threat to marine and freshwater ecosystems, agricultural production, groundwater, plant growth and even human and animal health. Disintegration of plastic products due to mainly biochemical or physical activities leads to the formation and existence of microplastics in significant amounts, not only in marine and freshwater environments but also in soils. There are several valuable studies on microplastics in soils, which have typically focused on environmental, chemical, agricultural and health aspects. However, there is also a need for the geotechnical engineering perspective on microplastic contamination in soils. In this review paper, first, degradation, existence and persistence of microplastics in soils are assessed by considering various studies. Then, the potential role of solid waste disposal facilities as a source for microplastics is discussed by considering their geotechnical design and addressing the risk for the migration of microplastics from landfills to soils and other environments. Even though landfills are considered as one of the main geotechnical structures that contribute to the formation of considerably high amounts of microplastics and their contamination in soils, some other geotechnical engineering applications (i.e., soil improvement with tirechips, forming engineering fills with dredged sediments, soil improvement with synthetic polymer-based fibers, polystyrene based lightweight fill applications), as potential local source for microplastics, are also mentioned. Finally, the importance of geotechnical engineering as a mitigation tool for microplastics is emphasized and several important research topics involving geotechnical engineering are suggested.

## 1. Introduction

Plastic products are being produced in increasingly vast amounts in a global scale. It is estimated that about 400 million tons of plastic production is made annually, and this amount is expected to more than double by 2050 [1]. Similar numbers were also reported by two recent publications: first one (359 million tons) supported by the European Parliament’s Policy Department for Citizens’ Rights and Constitutional Affairs [2], and the second one (368 million tons) supported by Association of Plastic Manufacturers in Europe [3]. These numbers indicate an alarming potential of global contamination for different ecosystems (marine, fresh water, soil, arctic) by plastics and their residues.

**Figure 1:**
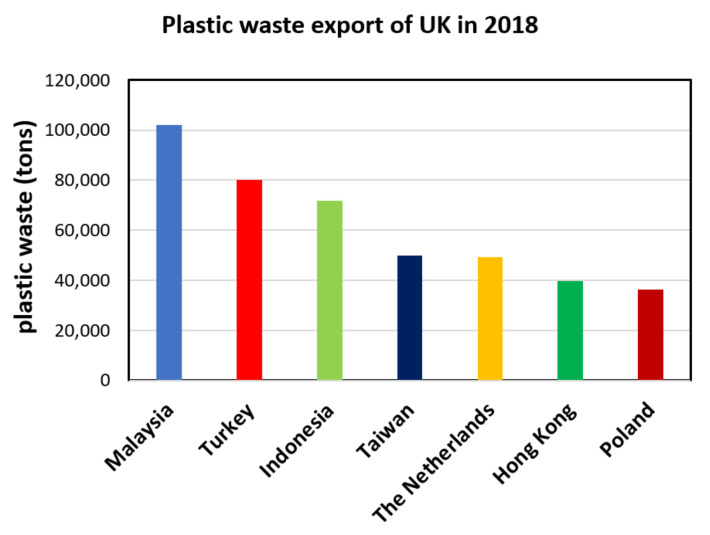
Top seven countries where the UK exported its plastic waste in 2018 (data from [6]).

For the past three decades, many countries including US, Japan, EU, Mexico etc. have exported their plastic waste to China and surrounding countries, which partially prevented the plastic debris going to solid waste disposal areas or incineration at those countries. However, in the last decade, China initiated restrictions on plastic waste importing policies, and finally banned the import of nonindustrial plastic waste in January 2018 [2,4]. Immediately afterwards, other Southeast Asian countries such as Malaysia rose noticeably as global plastic waste importers, but this was temporary, because such countries adopted restrictions on plastic waste importing policies as well. According to Zhao et al. [5], the center of gravity on global plastic waste trade is still evolving. For instance, the EU transformed from being a significant plastic waste exporter to being both a significant importer and exporter. Meanwhile, Turkey has become one of the growing plastic waste importers taking attention for the last few years. It suddenly entered the top 10 global plastic waste importers list in 2017, which was 10th in the list in 2017 with 26.19 × 10^4^ tons imported plastic waste and became 7th in 2018 with 43.69 × 10^4^ tons imported plastic waste [5]. In the same year (2018), Turkey became the second largest global importer of British plastic waste with 8 × 10^4^ tons [6] as shown in Figure 1, which increased to 21 × 10^4^ tons in 2021 [7]. However, it was dramatic to see that some of those imported plastic wastes were illegally dumped on fields and some burned, instead of being properly recycled [8]. In 2021, Turkey has initiated restrictions on plastic waste importing policies. Note that those reported random dump sites are not even Municipal Solid Waste (MSW) facilities, the engineering design of which would also be assessed considering microplastic contamination later in this paper.

Consequently, it is a big question not only for the major plastic waste importing countries, but also for all other developed/developing countries (which have vast amounts of plastic usage and debris) whether non-recycled plastic waste dumped illegally on fields or legally on MSW landfills significantly contaminate the soil environment by different means including macro, micro and nano plastics. For instance, the US Environmental Protection Agency reported that 35.68 million tons of plastic waste was generated in the US in 2018, and 26.97 million tons was accumulated in MSW landfills [9]. This indicates a very high percentage (i.e., 75.6%) of plastic waste dumped in MSW landfills, potentially threatening the soil and other environments in different ways, including microplastic contamination.

The term ‘Microplastic” was used for the first time in 2004 by Dr. Richard Thompson, a British marine ecologist, to refer to small plastic debris [1,10]. Today, upper particle size limit (i.e., ≤5 mm or 5000 μm) for classifying microplastics is widely used by researchers and scientists, however additional terminology (i.e., nanoplastics, mesoplastics etc.) and corresponding size ranges to define those terminology had been introduced. In general, microplastic size range is considered between 0.1 μm and 5000 μm, while plastic particles smaller than 0.1 μm are considered as nanoplastics in literature [11,12], even though different size ranges are also proposed both for nano and microplastics [2,13]. Considering the mass production of plastics accelerating rapidly (i.e., 10-fold increase by 2025 according to Pinto Da Costa et al. [2]), it is not surprising that UN Environmental Programme (UNEP) already announced microplastic contamination in marine environment among the top 10 emerging issues in 2014 [11,14,15].

**Figure 2:**
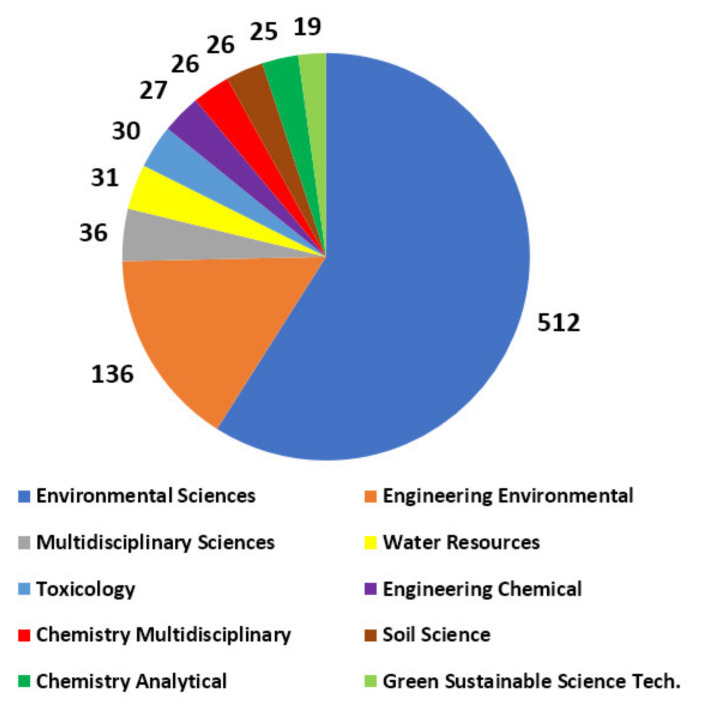
The distribution of the number of published studies on microplastics and soils in the Web of Science Core Collection database between January 2016 and October 2021 based on the top 10 disciplines.

Hence, microplastic contamination is already an increasingly ubiquitous problem which requires the cooperation of different disciplines (fields). However, it seems that there is a delay of global awareness between different disciplines regarding the microplastic contamination in different environments. For instance, Figure 2 gives the top 10 disciplines and the distribution of published studies on microplastics and soils in the Web of Science Core Collection database between January 2016 and October 2021 (some studies for 2022 were also available in the database but not included in Figure 2). Accordingly, the top 10 disciplines working on microplastics and soils for the past 6 years are Environmental Sciences, Environmental Engineering, Multidisciplinary Sciences, Water Resources, Toxicology, Chemical Engineering, Chemistry, Soil Science, and Green Sustainable Science Technology. Moreover, Figure 2 shows that there are only two Engineering disciplines (i.e., Chemical and Environmental) which show sufficient awareness (conducts considerable research) about microplastics and soils.

Authors of this study are Civil Engineers, and could definitely state that the current global awareness of the Civil Engineering community on microplastics is extremely limited, which is one of the main motivations of this paper. Geotechnical Engineering is one of the sub-branches of Civil Engineering, which deals with soils and their engineering behavior. Soils and their engineering behavior, (including but not limited to the concepts involving permeability, compressibility, shear strength etc.) are very important for many civil engineering projects such as foundation design of structures, design of tunnels, highways, bridges, ports etc. Nevertheless, geotechnical engineering (or geotechnics) can also act as an enhancing discipline to improve our understanding on microplastic contamination in soils from a different perspective.

The second section of this paper addresses the formation and existence of microplastics in soils, including the main degradation mechanisms influencing their presence, their concentrations in various soil environments (i.e., garden soil, landfill soil, agricultural soil, marine soil etc.) in different countries. Meanwhile, a geotechnical perspective is also expressed by the authors together with some key parameters (e.g., void ratio, hydraulic conductivity etc.) that could influence migration and spatial distribution of microplastics in soils. The persistence of microplastics in soils have also been considered through relatively long term field studies in literature, investigating the persistence of different polymers in real soil conditions. In the third section of the paper, geotechnical design of MSW landfills is discussed and their potential role as a source for microplastics and soil contamination are assessed. In the fourth section of the paper, some geotechnical applications, which are beneficial/smart from an engineering perspective, are reminded as additional potential microplastic sources in the long term. These geotechnical applications mentioned in the fourth part, are ground improvement with polymer-based fibers, ground improvement with waste tire-chips, lightweight polystyrene foam applications, reclamation fills with microplastic contaminated dredged soils. In the last (fifth) section of the paper, authors explained some future research ideas regarding how to benefit Geotechnical Engineering as a mitigation tool for reducing the microplastic contamination in soils and gave concluding remarks.

## 2. Formation and Existence of Microplastics in Soils

Even though microplastic contamination affects various ecosystems including marine [16] and freshwater environments [17], soils [18] etc., terrestrial soils are perhaps the relatively less studied one among the three. Nizzetto et al. [19], Zhang et al. [20], Zhu et al. [21], Da Costa et al. [22], Möller et al. [23] and Wang et al. [24] have all explicitly mentioned that research on microplastics in soil environment is still relatively limited in number and content compared to other environments. Authors could list several reasons for such an observation including but not limited to: (1) relative delays for the global awareness between different disciplines on microplastic contamination (e.g., Environmental Sciences vs. Civil Engineering; Chemistry vs. Geology etc.), which was mentioned previously (Figure 2); (2) lack of multidisciplinary research on microplastics in soils between soil related disciplines and others (e.g., bwtn. Environmental Sciences, Chemistry and Geotechnical Engineering); (3) historical/gradual development of microplastic awareness, which originally initiated for the marine environment [10].

Nevertheless, research and relevant studies on microplastics in soils also gained a momentum in the last few years as well, including the analytical methods for microplastic sampling and determination in soils [22,23,25]; their migration, ecological and environmental risks [5,18,26]; their transfer and accumulation in agricultural soils [27,28,29]; their effects on soil quality and function [30,31].

The mentioned studies on microplastic-soil interaction understandably focused mostly on agricultural, environmental and health aspects, where there is little-to-no engineering perspective involved. For example, De Souza MacHado et al. [30] conducted an experimental study and observed that microplastics influence the bulk density and water holding capacity of soils. They interpreted the results from an agronomical perspective, since there is a good correlation between soil bulk density and rootability. More specifically, a reduction in soil bulk density would normally imply better rootability, due to increased soil porosity helping root growth [30,32]. However, as also questioned by De Souza MacHado et al. [30], such a decrease in bulk density due to microplastics could be misleading for rootability. Since it is unclear how much the soil porosity actually increased in their experimental study considering that the densest microplastic polymer used in their study (polyester) is still considerably lighter (~1370 kg/m^3^) than their control soil (~1439 kg/m^3^). More explicitly stated, the observed decrease in bulk density of soil due to microplastic addition in the experiments could be more affected by addition of a lighter material than the soil itself, instead of increasing soil porosity. From a typical geotechnical engineering perspective, bulk density and porosity intuitively remind shear strength and settlement/compressibility characteristics of a soil [33,34]. Both shear strength and compressibility are two very important concepts related with many aspects in civil/geotechnical engineering, including slope stability [35], foundation design of engineering structures [36], dredging and reclamation works [37], landfill design [38] etc. Though void ratio (e) (which is the ratio of the volume of voids to the volume of solids in a soil matrix) is a more popular parameter than the porosity (n) (which is the ratio of the volume of voids to the total volume in a soil matrix) in geotechnical research and practice. Nevertheless, changes in soil density due to microplastic contamination is not expected by the authors to influence its overall engineering behavior in terms of settlement or shear strength, unless extreme levels of contamination exist in the soil.

### 2.1. Degradation Processes

Several different degradation processes exist for microplastic formation, which are typically known to have a slow rate depending on the ambient conditions. The main mechanism of degradation could involve chemical, physical, biological or a combination of those processes. More specifically, polymeric degradations playing role on microplastic formation can be listed as thermal, mechanochemical, ozone-induced, biological, photo-oxidative, and catalytic types of processes [39,40], and interestingly, these processes seem to influence their toxicity as well [26,41]. O’Kelly et al. [15] stated that ozone-induced degradation, biodegradation and photo-oxidative degradation are the main degradation types contributing to breaking down the “macro” and “meso” plastics and consequently forming microplastics in soils.

Singh and Sharma [40] discussed the detailed mechanisms of the above-mentioned degradation processes. Accordingly, photo-oxidative degradation is a decomposition process which occurs by the absorption of the UV and visible lights by the polymers. Then, degradation and oxidation reactions start, which are determined by the groups attached to the polymer and/or the impurities in it.

Ozone-induced degradation is caused by the ozone in the air, even though it is present in very small concentrations. Nevertheless, it is enough to accelerate the aging of the polymeric materials. This phenomenon is followed by the intensive formation of oxygen-containing compounds, as well as changing the molecular weight and the mechanical and electrical properties of the materials. Ozone-induced degradation occurs by the attack of the ozone molecule to the unsaturation in unsaturated polymers where the reaction propagates in three main steps; firstly, the formation of ozone olefin adduct, secondly the decomposition of the primary ozonide to carbonyl compounds and a carbonyl oxide, and thirdly the fate of the carbonyl oxide [40].

Biodegradation of polymers is typically caused by the available microorganisms and enzymes in soils. Examples to such microorganisms could be different fungi and/or bacteria, while different enzymes (e.g., polyurethanase, lipase etc.), could contribute to the biodegradation process [40,42,43]. For example, Crabbe et al. [44] studied four fungus types isolated from soil (Curvularia sene galensis, Fusarium solani, Aureobasidium pullulans and Cladosporium sp), and concluded that they could biodegrade ester-based polyurethane. Yoshida et al. [45] discovered a novel bacterium (Ideonella sakaiensis), which could use PET (polyethylene terephthalate) as an energy and carbon source for living (i.e., it efficiently biodegrades PET). In a recent study, Feng et al. [46] investigated the biodegradation mechanism of PET with the molecular dynamics and quantum mechanics/molecular mechanics approaches. Their study focused on the two enzyme system (IsPETase and IsMHETase) in the mentioned novel bacterium (Ideonella sakaiensis). Orhan et al. [47] studied biodegradation of plastic compost bags under controlled soil conditions. In their study, Orhan et al. [47] investigated the response of supposedly degradable and non-degradable low (LDPE) and high-density polyethylene (HDPE) in soil mixed with 50% (*w*/*w*) mature municipal solid waste compost supplied from municipal refuse and mentioned that the rate of polymer biodegradation is affected by environmental factors such as moisture, temperature and biological activity. Similar environmental factors were also mentioned by Kliem et al. [42] for the biodegradation of different polymers. Moreover, there is another aspect of biodegradation in terms of terminology. As Kyrikou and Briassoulis [48] discussed in detail, a biodegradable polymer is expected to leave no harmful substances in the environment, which means that it should be entirely converted to carbon dioxide, water, mineral and biomass, without any ecotoxicity. Kyrikou and Briassoulis [48] also explained that many polymers that are considered as “biodegradable” are indeed degradable depending on environmental conditions and better be called “hydro-degradable”, “photo-degradable” or “oxo-degradable”.

It is also worth to mention that photo-oxidative, and ozone-induced degradations may also promote the biodegradation potential of polymers due to breaking of polymer chain and increasing the surface area for colonization of microorganisms and/or by decreasing molecular weight [15,40,49,50].

### 2.2. Existence of Microplastics in Soils

The existence of microplastics in soil environment is a globally emerging issue, which should alert not only the scientific community, but also the public and policy makers. Several studies reported microplastic concentrations available in different soils worldwide.

Huerta Lwanga et al. [51] investigated surficial samples (0–20 cm) from 10 home garden soils in Mexico from sites selected from similar ethnic and economic demographics, and reported microplastic concentration of 870 ± 1900 ptcl./kg (particles per kilogram of soil). Zhang and Liu [29] investigated the distribution of plastic particles over four agricultural sites involving cropped soils and another site at riparian forest buffer zone in China. All soil samples were taken from surficial layers (0–10 cm), and an average of 18,760 ptcl./kg were reported, in which 95% of the particles were in the microplastic range based on their classification (i.e., 0.05–1 mm). Zhang and Liu [29] also noted that microplastic concentrations were higher for the agricultural soils in their study compared to the forest buffer zone soil.

Crossman et al. [28] studied microplastic concentrations in soils between 0–15 cm depth at three agricultural sites in Ontario, Canada. Accordingly, average microplastic concentrations for the three sites vary significantly (i.e., 18 ptcl./kg ±22.2%; 187 ptcl./kg ± 53.1%; 541 ptcl./kg ± 56.4%, respectively). van den Berg et al. [52] inspected soils from 16 agricultural sites in Spain, and found that average microplastic concentration is 930 ± 740 ptcl./kg for light density microplastics (i.e., ρ < 1 g/cm^3^), while it is 1100 ± 570 ptcl./kg for high density microplastics (i.e., ρ > 1 g/cm^3^) based on surficial samples (0–30 cm).

In a recent study, Dahl et al. [53] investigated the contamination of seagrass soils (i.e., marine soils) at three sites along the Spanish Mediterranean coast, and concluded that microplastic contamination was negligible until 1975s, then increased dramatically. The samples were taken from shallow depths from the soil surface (0–15 cm) and the concentrations change (between 68 and 3819 ptcl./kg) depending on the site. However, Dahl et al. [53] stated that there is a strong relationship between the intensification of the agricultural industry at a particular region and the microplastic concentrations in the soils.

As expected, the studies mentioned above reveal that concentrations and existence of microplastics in soils vary with country (e.g., Mexico, China, Canada, Spain), location and characteristics of the site (i.e., garden soil, landfill soil, agricultural soil, marine soil etc.), regional industrial practices (e.g., agricultural applications) and possibly from many other interlinked sub-factors such as the level and quality of the waste solid/water treatment plants, regional population intensity, economic level and usage of plastic involving products etc.

Another observation from the studies reviewed above is that they all focused on the microplastic existence within the first 20 or 30 cm below the ground surface. This is understandable, considering that soil life is more active in those depths and typical rooting and ploughing depth do not exceed 30 cm [52]. However, this depth range (0–30 cm) is very shallow from a geotechnical engineering perspective. Hence, more studies are needed to quantify the existence of microplastics at greater depths from the ground surface. Permeability or hydraulic conductivity (k) is one of the key parameters in geotechnical engineering (also in geology and hydrogeology), which indicates the ability of water and other fluids to flow through the voids between the soil grains [54]. Hydraulic conductivity is a function of different factors including but not limited to density (ρ_soil_) and void ratio (e) of the soil, viscosity of the pore fluid (e.g., clean or contaminated water), type of the soil (e.g., clay, silt, sand), effective pore size between the soil grains etc. [54,55]. Nevertheless, authors think that hydraulic conductivity (k) could be among the key parameters for spatial distribution of microplastics in soils both in horizontal and vertical directions, therefore deserves attention during future research on microplastics in soils.

Several studies are available in literature about the migration of microplastics in soils. O’Connor et al. [56] conducted an experimental study about the vertical migration of microplastics in sands from wetting-drying cycles perspective. They investigated the mobility of five different microplastics having different sizes and densities, which consist of polypropylene (PP) and polyethylene (PE) particles. They found that maximum penetration depth of microplastics through sand almost linearly increases with the number of wetting-drying cycles, and as the microplastic size becomes smaller, its mobility in the soil also increases. O’Connor et al. [56] also mentioned that microplastic concentration at the surface and volume of infiltration liquid had only negligible or weak effects on depth of migration. Moreover, they forecasted the long-term penetration depths based on weather data of 347 Chinese cities and their experimental model. Accordingly, they estimated an average penetration depth of 5.24 m in the long term (≈100 years). The study of O’Connor et al. [56] is certainly very valuable in terms of the influence of wetting-drying cycles on microplastic penetration in sands. However, from geotechnical engineering view, sands are only one of the several soil types (e.g., clays, silts, gravels and their mixtures) in engineering classification, hence the penetration depth of microplastics can be expected to change with various other factors explained before (e.g., soil type, void ratio, hydraulic conductivity etc.).

De Souza MacHado et al. [30] also conducted an experimental study on loamy sand, where they measured the hydraulic conductivity (though in a different way compared to the ASTM standards used in Civil Engineering). They stated that the existence of microplastics in sand did not significantly change the hydraulic conductivity of the soil. From geotechnical point of view, sandy soils typically have quite high hydraulic conductivities ranging from 1 to 10^−2^ cm/sec for clean coarse sands, and from 10^−2^ to 10^−4^ cm/sec for clayey sands [54,57]. In fact, De Souza MacHado et al. [30] also acknowledged this aspect and wrote that the hydraulic conductivity of the sandy soil that is used in their experimental program could be high enough to be unaffected by the microplastic concentrations and adopted k measurements in their study.

Wu et al. [58] ran column experiments to investigate the vertical migration response of polystyrene nanoplastics in three natural soils from China with contrasting physicochemical properties (e.g., salt composition, ionic strength, zeta potentials etc.). They reported that soil mineralogy and pH influence the migration of nanoplastics in soil medium, where the migration of nanoplastics was also reported to be sensitive to ionic strength and cation type.

Consequently, there have been several valuable studies which investigated the existence and migration of microplastics in soils from different aspects. From geotechnical perspective, authors want to emphasize/remind the importance of following factors/research gaps for future studies: (1) distribution and migration of microplastics in non-shallow soil depths (i.e., ≥30 cm); (2) influence of hydraulic conductivity (k) for spatial distribution and migration of microplastics in soils both in horizontal and vertical directions; (3) consideration of the influence of different soil types used in engineering classification (e.g., clay, silt, sand, gravel and their mixtures) on microplastic distribution in soils. All the three aspects listed above deserve further systematic multi-disciplinary research involving geotechnical engineering.

### 2.3. Persistence of Microplastics in Soils

**Figure 3:**
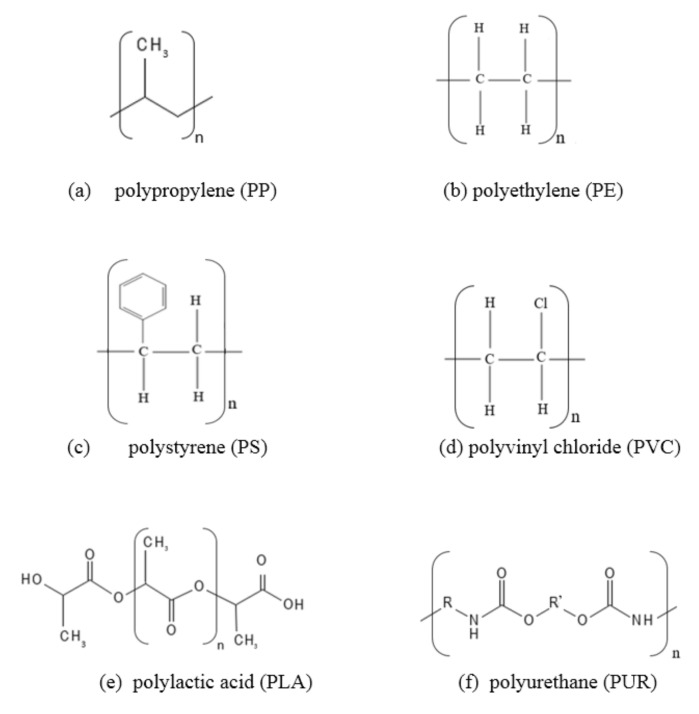
Molecular structures of some of the commonly encountered polymers in soils (**a**) polypropylene (PP), (**b**) polyethylene (PE), (**c**) polystyrene (PS), (**d**) polyvinyl chloride (PVC), (**e**) polylactic acid (PLA), (**f**) polyurethane (PUR).

It is known that microplastics are quite persistent in soils (i.e., their degradation process takes a very long time). Molecular structures of some of the commonly encountered polymers in soils are shown in Figure 3, which would also be addressed in different parts of this paper based on different studies.

Cooper and Corcoran [59] investigated plastic particles from 5 beach soils at Kauai Island, Hawaii. They warned that microplastics formed by disintegration of macroplastics remain in the environment almost indefinitely, which cannot be tolerated simply by using more rapidly-degrading polymer types, especially with the accelerating trend of plastic usage. A parallel observation was also made by Krueger et al. [60], who mentioned that the present-day synthetic polymers are quite persistent against biodegradation (some having degradation period of decades or even centuries), and this, in turn cannot counteract with the overwhelming pollution with plastics. Krueger et al. [60] also compiled several laboratory studies about the biodegradation of synthetic polymers in different environments, including soil, marine conditions, and mentioned that most plastics are quite recalcitrant (with low reaction rates) even under optimized laboratory conditions. More importantly, Krueger et al. [60] claimed that published laboratory studies could be strongly biased to successes obtained under optimized laboratory conditions, which have limited transferability to real environments.

Fortunately, there are also few relatively “long” term field studies in literature investigating the persistence of different polymers in real soil conditions. Briassoulis et al. [61] buried low-density polyethylene (LDPE) mulching films in an agricultural soil (after being used for one watermelon cultivation) to simulate and observe the long-term degradation behavior in field conditions. In their experimental study, Briassoulis et al. [61] buried pro-oxidant added mulching films for 8.5-year period in soil. Pro-oxidants are special additives, which involve mainly carbonyl groups and metals blended with different ingredients (e.g., cobalt acetylacetonate, magnesium stearate etc.) which accelerate the breakdown of polyethylene [61,62]. Briassoulis et al. [61] reported that after 8.5 years staying in soil, buried low-density polyethylene mulching films were recovered almost intact with no disintegration observed, which implies the persistency of even macroplastics (i.e., PE in that study) in soils. Study of Albertsson and Karlsson [63] also gave similar results, who investigated the degradation behavior of LDPE film in laboratory conditions for a 10-year cultivation period with soil.

Otake et al. [64] examined the biodegradation of different polymers, when buried in soil for about 32 years. They determined different synthetic polymer types in a Japanese garden soil from 10 and 50 cm depths from the ground surface, including LDPE, polystyrene (PS), polyvinyl chloride (PVC), and urea formaldehyde (UF) resin buried in soil between 32 and 37 years. They reported that for PVC, PS, and UF resin, no biodegradation was observed after over 32 years, however LDPE samples have shown signs of degradation. Otake et al. [64] also mentioned that the signs of degradation were more visible for samples collected from shallow depths (~10 cm) than the ones collected from relatively deeper levels (~50 cm), possibly because of having more aerobic activity at shallow depths. Higher persistence of microplastics in deeper soil layers is due to smaller microbial population available, compared to the shallow depths, which would reduce their degradation potential. This observation makes the research topic of “distribution and migration of microplastics in non-shallow soil depths” mentioned by the authors in previous Section 2.2 also important from persistence point of view as well.

Tabone et al. [65] investigated the sustainability metrics (which include atom economy, mass from renewable sources, biodegradability, percent recycled, distance of furthest feedstock, price, life cycle, health hazards, and life cycle energy use) of 12 polymers. Accordingly, “biodegradable” polymers (e.g., polylactic acid (PLA), polyhydroxyalkanoate (PHA)) are listed on top of the green design rankings of their study, however those polymers also exhibit relatively large environmental impacts from production [65]. Since PLA (Figure 3e) is being increasingly used in short shelf-life products (i.e., in compostable food-packaging films, bags etc.), wastes involving PLA increase in the environment [66]. Karamanlioglu and Robson [66] investigated the degradation behavior of PLA in commercial packaging buried in soil and compost for a temperature range (i.e., btwn., 25 °C and 55 °C). Accordingly, no change in tensile strength or molecular weight was observed after 1 year at relatively low temperatures (i.e., 25 °C and 37 °C), which implies a problem for PLA persistence in soils. However, they observed that at elevated temperatures (i.e., 50 °C) microbes enhanced the biodegradation process of PLA, which indicates the importance of soil temperature on biodegradation.

## 3. Solid Waste Disposal Facilities and Their Potential Role as a Source for Microplastics

### 3.1. Solid Waste Disposal Facilities and Their Geotechnical Design

Rapid population growth and industrialization are the two key factors that contribute to environmental pollution. Furthermore, production diversity and changes in the consumption habits result in an increase in the amount of waste materials. These waste products are mainly composed of municipal solid wastes, which are the waste materials that human beings use and throw away every day. Although the solid wastes are partially recycled or burnt, it is not possible to eliminate all the particles of these wastes [67,68]. Therefore, it is aimed to store the municipal solid wastes (MSW) in specific waste disposal facilities that are called MSW landfills or simply landfills in order to minimize their harmful effects on the environment by isolating them from the subsoil environment and the groundwater.

In order to catch and evacuate the surface flow, a drainage system has to be installed and the landfill has to be built in accordance with the natural environmental conditions by growing plants on top of it or making a social area for the public [69]. Separation and classification of the solid wastes is a crucial step for providing some of the wastes to be recycled and reducing the use of the natural resources and as a result, for preventing environmental pollution. Considering that the separated solid wastes also have harmful effects on the environment and human health, these wastes have to be stored in the landfills that are designed for various purposes. Determination of the engineering properties of the soil profile and the groundwater conditions, measurement of the piezometric heads in the aquifers, the hydraulic conductivity of the soil, and characterization of the geochemical conditions play important roles in the selection of the location of a landfill [70].

The base and the sides of a landfill have to be covered with a barrier or liner material in order to control/prevent soil and water contamination. For this purpose, compacted clay liners (CCLs) or geosynthetics are generally preferred to be used as the barrier materials in landfills [71]. The main aim for placing the liner material in a landfill is to prevent or control the permeation of the leachate through the barrier to the subsoil and groundwater. Compacted clay liners are composed of natural clay deposits and the hydraulic conductivity of the compacted clay depends on the clay mineralogy, void ratio and water content of the clay during compaction and the method of compaction. CCLs are typically compacted at water contents greater than the optimum water content (w_opt_) obtained from Proctor Compaction Tests in order to achieve smaller permeability for the liners. As a result, the hydraulic conductivity of the CCL is correlated with the void ratio. The CCL that is selected to be used in a landfill should satisfy several geotechnical criteria. For instance, the hydraulic conductivity of CCL, k ≤ 10^−9^ m/s; the dry weight percentage of the fine soil particles that pass through 0.075 mm sieve (No., 200) ≥ 50%; the plasticity index ≥ 7–10%; the dry weight percentage of the soil particles that remain on 4.75 mm sieve (No. 4) ≤ 20% [72,73].

Geomembranes and geotextiles are the two major geosynthetics that are used for various purposes in landfills. The geotextiles are flexible textile materials with synthetic fibers and they typically provide filtration in a landfill. The geomembranes are thin sheets of impervious plastic materials. A geomembrane layer is typically placed over the CCL to provide imperviousness against leachates [72]. A geomembrane is the additional lining layer over a CCL that is used for enhancing the barrier capacity of the clay liner in a waste disposal facility. However, the geomembrane can easily be damaged or punctured by a sharp particle that can be found in a solid waste. Moreover, installation damages on geomembranes are not uncommon if the construction quality assurance and quality control are not strictly applied [74,75]. If such damages occur, leachate involving microplastics can seep into the underlying CCL.

**Figure 4:**
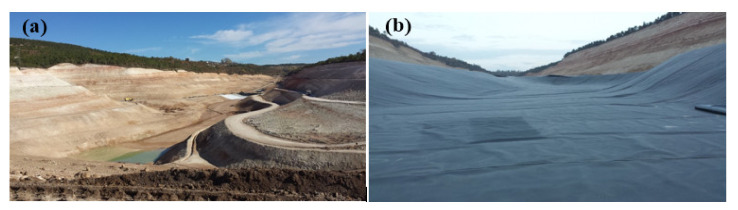
Waste disposal area in Kütahya, Turkey: (**a**) without a barrier material; (**b**) with the barrier material that consisted of a geomembrane-laminated GCL.

A geosynthetic clay liner (GCL) is a lining and barrier material that consists of a thin bentonite layer sandwiched between two geotextiles [72]. In recent years, the design engineers have preferred to use geosynthetic clay liners (GCLs) as an alternative to the CCLs as the barrier material in the waste disposal facilities [76]. Moreover, the GCLs have some advantages over the CCLs such as having lower hydraulic conductivity (<10^−10^ m/s), lower thickness (4–10 mm), lower cost, less labor work and faster installation [72,77,78]. The most critical component of a GCL that determines the hydraulic performance of the barrier material is the bentonite layer [79,80,81]. A waste disposal area in Kütahya, Turkey before and after the placement of a barrier system that was composed of a geomembrane-laminated GCL is shown in Figure 4.

**Figure 5:**
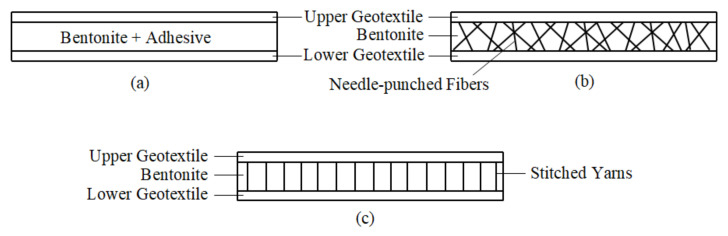
Cross-sectional views of GCL types: (**a**) Adhesive-bonded GCL; (**b**) Needle-punched GCL; (**c**) Stitch-bonded GCL.

According to their manufacturing process, the GCLs can be classified into three groups. The adhesive-bonded GCL is composed of a bentonite layer attached to the upper and lower geotextiles with a water-soluble adhesive without any reinforcement. The needle-punched GCL is manufactured by punching the needle-like fiber particles from the upper geotextile through the bentonite layer to the lower geotextile. Due to the reinforcement provided by the needle-punching process, the migration of the bentonite from the GCL is mostly prevented in this type. The stitch-bonded GCL is another reinforced GCL type. For the stitch-bonded GCL, the upper and lower geotextiles are stitched together with parallel oriented yarns by keeping the bentonite layer inside the GCL [71,72,75]. The cross-sectional views of the three different GCL types mentioned are shown in Figure 5.

**Figure 6:**
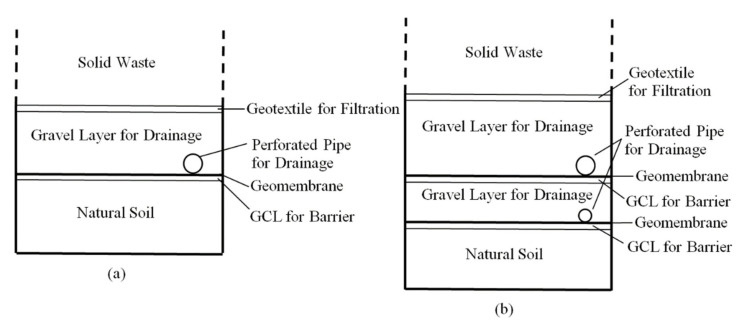
Cross-sectional view of landfill liner systems: (**a**) Single-liner system; (**b**) Double-liner system (Upper layer for leachate collection and lower layer for leak detection).

In a typical landfill liner system (shown in Figure 6), first, the GCL which is used as the lining and barrier material is placed over the subsoil (natural soil). Then, a geomembrane layer is preferred to be used between the drainage layer and the GCL in order to protect the lining material against possible sharp gravel or solid waste particles that might puncture or tear the GCL as well as acting as an impermeable interface between the drainage layer and GCL. The gravel layer with a minimum thickness of 30 cm that is placed over the geomembrane, acts as the drainage layer of the landfill.

The efficiency of the operation of a landfill depends on the installation of a proper drainage system that collects and removes the leachate from the landfill properly [70]. The selection of the barrier materials and the transmission pipes for the leachate drainage are the crucial steps for the design of the landfill drainage systems. In order to collect the leachate, the drainage system has to be designed with an inclination of 0.5–1% [71,82,83]. The main reason for this inclination is to provide the collection of the leachate by creating the needed hydraulic head on the barrier material. The capacity of the drainage system plays the critical role for determining the exact length and inclination of the drainage pipes. The perforated pipes, which are installed in the leachate collection system, are used for removing the leachate from the landfill. As a result, the leachate is sent to the surface of the landfill for wastewater treatment [67,72,73]. The geotextile that is placed over the gravel drainage layer serves as a filtration layer as can be seen in Figure 6. The geotextile is used for preventing the clogging of the drainage layer with the particles of the solid waste. Finally, the solid waste is dumped into the landfill and can be stored in a single-liner system as shown in Figure 6a.

The geotechnical design of a landfill determines the performance and the functionality of the liner system. The construction and demolition debris are typically buried in landfills that have single-liner systems. However, single liners are not preferred to be used to store municipal solid wastes. A double-liner system is composed of two single liners as shown in Figure 6b. In a double-liner system, the upper liner is used for collecting the leachate whereas the lower liner serves as a leak-detection system. The gravel layer in the leachate-collection system has a height of at least 30 cm while the height of the gravel layer in the leak-detection system is half of the height of the upper gravel layer that functions for drainage [72].

Double-liner systems are generally used in both municipal solid waste and hazardous waste landfills [69,72]. Although a double-liner system has more barriers and lining components than a single-liner system as shown in Figure 6, there is no guarantee that the leachate will completely remain above the natural subsoil environment. Thus, there is still the risk for the contamination of the soil and the groundwater, which is also a potential threat for different levels of microplastic migration into the surrounding soil environment. In fact, the levels of microplastic contamination in soils at the vicinity of different landfills could be an important research topic, to be further studied.

### 3.2. The Risk of Microplastic Migration from Landfills to Soil and Other Environments

Plastics cover a significant amount of the solid wastes and 79% of the plastic wastes are either stored in landfills or released to the natural environment [84]. 21–42% of the global plastic wastes that are not recycled and not burnt, are stored in landfills [85]. Approximately 12,000 million tons of plastic wastes are estimated to be stored in landfills or released to the environment in 2050 [84]. Isolating the bottom and sides of a landfill with a barrier system could prevent or at least limit not only the leakage of the leachate from the solid wastes but also the migration of the microplastics to the subsoil and the groundwater.

The plastic wastes that are stored in the landfills may undergo both physical and biochemical changes due to many factors including biodegradation (explained in Section 2.1), drastic changes in temperature (temperature may rise up to 60–90 °C) and acidity (pH fluctuations between 4.5 and 9), CH_4_ or CO_2_ generation, high salinity or high pressure and compaction [86,87,88,89]. As a result of these changes, the plastic wastes can be fragmented into smaller pieces that are classified as microplastics. The soil and the groundwater can be contaminated due to the possible transfer and migration of these microplastics from the landfill to the subsoil environment. The lack of sufficient liner systems for abandoned waste dumps or defects/liner damages in landfills at different countries might cause environmental problems due to the discharge of the leachate that carries out the microplastics generated from the plastic wastes into the soil.

Approximately 52 million tons of municipal waste materials were stored in landfills of the EU countries in 2018 [90]. According to Xu et al. [90], this amount corresponds to 10.4 million m^3^ of leachate formation and 3.03 billion in microplastic release to the environment. Considering that these values were only obtained in the EU countries, the amount of the microplastic release for the whole world would be much higher. In another study, the average leachate formation from 1000 kg of solid waste that was deposited in landfills in 30 different cities of China was measured as 1.3–3.2 m^3^ [91].

He et al. [85] also detected microplastics in all of the 12 leachate samples taken from six different landfills in China. Polypropylene (Figure 3a) and polyethylene (Figure 3b) were found to be the two major polymer types among the microplastic particles in their study. Approximately 77.5% of the microplastics had a particle size between 0.1–1 mm and the concentration of the microplastics in the landfill leachates was within the range of 0.42–24.58 ptcl./L (particles per liter). Meanwhile, the concentration of the microplastics in the leachate of landfills from Northern Europe was determined only up to 4.51 ptcl./L in the study of Van Praagh et al. [92]. This difference in concentrations could be due to the strict environmental regulations, lower population and environmental awareness of the Northern European countries when compared with those of China. Similar to He et al. [85], Su et al. [93] also reported that the most widely encountered polymer types of the microplastics in a landfill leachate from China were determined as polyethylene and polypropylene, whereas the study of Van Praagh et al. [92] has shown that polyurethane (Figure 3f) and polyethylene (Figure 3b) were the two dominant polymers in landfill leachates in the North European countries. The particle size of the microplastics from the landfill leachates in the North European countries was within the range of 0.05–5 mm. Furthermore, the dominant shape of the microplastics in several leachate samples was angular with sharp edges [85,94].

Landfill leachate might also contain hazardous substances such as Bisphenol A (BPA). Due to hydrolysis, diffusion and physicochemical processes, BPA can easily be released from plastic materials to the environment [95,96]. As the plastics are decomposed into microplastics and the microplastics are carried with the leachate flow, the BPA can also be mixed with the soil and the groundwater. The concentrations of BPA and microplastics were measured almost the same in a study conducted by Narevski et al. [94] in which leachate samples, that were taken from three landfills in Southeastern Europe, were analyzed. The results indicated that the concentration of the microplastics was within the range of 0.64–2.16 mg/L whereas the BPA concentration was found to be 0.70–2.72 mg/L. Due to their high absorption capacity, the microplastics may easily absorb toxic organic substances [97] and increase the heavy metal concentration in soils [98]. Soils involving these microplastics, that are carried with the leachates seeping into the soil in the vicinity of landfills, are sometimes used in agricultural practices and the toxic substances that are absorbed by the microplastics might quickly deteriorate soil health and hinder plant growth.

There are also relatively limited amount of studies that indicate the occurrence of microplastics in soil layers and groundwater beneath landfills. For instance, the wrinkles in the geomembrane liner systems could cause the leachate with microplastics to migrate through the barrier material to the subsoil [99]. Manikanda et al. [100] detected microplastics in the groundwater from the subsoil beneath municipal solid waste landfills in South India. The microplastic concentrations in the groundwater were measured as 2–80 ptcl./L and the main polymer types of the microplastics were classified as polypropylene (Figure 3a) and polystyrene (Figure 3c). Microplastics were also observed in several soil samples from a sanitary landfill in Bangladesh. The polymer types of the microplastics were expressed as polyethylene and cellulose acetate. The particle diameter of the microplastics detected in the soil beneath the landfill was within the range of 0.001–2 mm [101].

Both leachate and soil samples were taken from several landfills in Thailand [102]. The soil samples were collected from the upper layer of the subsoil beneath the landfill bottom with a depth of 10–20 cm by a hand auger. It is interesting that the microplastic concentration was found to be higher in soil than in leachate. According to Puthcharoen and Leungprasert [102], the soil samples involve microplastics approximately 1500 ptcl./kg while there were only 20 ptcl./kg microplastics in the leachate samples. The main polymer types of the microplastics obtained from both the soil and the leachate samples were polyethylene, polypropylene and polyethylene terephthalate. The dominant microplastic patterns were granules and films for both the soil and the leachate samples. The other microplastic patterns observed in the soil and leachate samples were spheres, irregulars and fibers. The granule, sphere and irregular-shaped microplastics were reported to be mainly decomposed from plastic food storage containers and water bottles. On the other hand, plastic packages and bags were the main source of the film-shaped microplastics. Fiber-shaped microplastics were mainly formed by the decomposition of synthetic fibers from synthetic clothes [103]. In another study, the solid waste samples taken from different landfills in Shangai, China were examined and according to the results, the older landfills contained a wider range of microplastics in terms of shape. However, the contribution of the landfill age to the microplastic shape in leachates was not as critical as that of in solid wastes [93].

The main agents that contribute to the horizontal transportation of the microplastics are surface run-off, wind erosion, flooding and animal scavenging [15,19]. Lighter microplastics can be much easily transported by wind from the surface of the soils to other lands or water resources, while the denser microplastics are more likely to vertically migrate into soil [104,105].

Some studies indicate that the microplastics can be also transferred from landfills to some water resources or other lands. For example, microplastics were detected in a river that was close to a landfill located around a coastal area [106]. The investigations showed that the landfill leachate had carried out the microplastics to the river. According to some studies, the landfills that are built on coastal areas or beaches might collapse due to erosion. Global warming that leads to increases in sea levels is the main reason for such erosion triggered failures. As a result, the collapsed landfills might cause the scattering of the microplastics to the sea and contamination of the water [107,108]. Due to the atmospheric transport of the lighter microplastics from landfills to either other terrestrial regions or any sea, lake or river horizontally, there is the risk for the human beings to inhale the microplastics as well [109]. When inhaled, microplastics may reach the lungs and stomach, and threaten human respiratory and digestive systems [110,111].

As discussed here, the microplastic transfer from landfills to soils, water resources and atmosphere has been evaluated in different studies. According to these studies, the leachates are the main agents that carry microplastics from landfills to the soil deposits vertically. Although the particle size of the microplastics that were collected from both leachates in landfills and soil samples beneath landfill bases varied in a wide range, the main polymer types of the microplastics were found to be polypropylene and polyethylene as shown in Figure 3. Furthermore, the primary sources for the formation of the microplastics in the landfills were plastic food storage containers, plastic packages and plastic bottles. The microplastics could also be carried from landfills to water resources such as rivers, lakes or seas, to terrestrial regions/soils far away from the landfills or to the atmosphere by mainly wind and surface run-off. The microplastics in leachates or soil beneath the landfills might also contain hazardous or toxic substances. As a result, microplastics can be easily transferred to the soil from a landfill without a barrier material or with a barrier material that is damaged or not properly designed.

## 4. Some Geotechnical Engineering Applications as Potential Source for Microplastics

Landfills are considered by the authors as one of the significant sources of microplastic contamination in soils, which deserves further attention. However, there are also other geotechnical engineering applications, where polymer-based materials (e.g., tire chips) or already contaminated soils (e.g., dredged soils) are used. Such applications inherently have the potential to generate microplastics as well, even though their scale of contamination is expected to be smaller than the ones due to landfills. Some of these applications are explained below:

### 4.1. Soil Improvement with Tire Chips

Tire chips are the shredded waste tires obtained from the abrasion of road vehicle and airplane tires. Tire wastes are one of the most significant microplastic sources for the migration of the microplastics to the soil. As a result of the disintegration of the shredded waste tires, microplastics can enter the soil easily. The global emission of microplastics due to the abrasion of road vehicle tires is approximately 0.81 kg/year per capita [112]. Moreover, the most abundant source of annual microplastic mass emission per capita for the EU countries was found to be the tire wear with 1784.8 g.cap^−1^ a^−1^ [113]. Microplastics formed by the disintegration of the waste tires were also observed in various landfill leachate samples [114].

On the other hand, waste tires are also used as geomaterials for enhancing the shear strength of soils in civil engineering projects. In order to reduce the negative impacts of the shredded waste tires on the environment and seeking for economical engineering solutions, soil deposits mixed with tire chips are used in various geotechnical applications including road embankments, pavement subgrades and backfills of retaining structures [115,116,117,118].

Singh and Sonthwal [117] and Solanki et al. [118] added tire chips to clayey soils and the tire chip addition in these studies led to an increase in the shear strength of the clay. Tire chips were also mixed with sandy soils in various studies. Daud [115] reported that the shear strength of a sandy deposit was increased by tire chip addition with an observed increase in the internal friction angle of the soil. Al-Neami [119] conducted California bearing ratio (CBR) tests on tire chip-sand mixtures and the results indicated that tire chip addition with a content of 8% by dry weight caused 1.6 times higher load bearing capacity than the sand without any tire chip addition. CBR and unconfined compression tests were performed on tire chip-cement-soil mixtures, and according to the results, both the shear strength and the bearing capacity of the soils increased due to tire chip addition [117,120].

### 4.2. Dredge Sediments from Water Resources Used as Filling Materials

Dredge sediments are typically carried from the bottom of rivers, lakes, harbors or any other water resources to the target sites and dumped to fill lowlands. The dredge sediments are mainly used for urban soil reconstruction, shoreline stabilization, coastal land reclamation, upland placement for agriculture, as fertilizers in farmlands or as covers for landfills and mine tailings dams [37,121,122,123]. Due to the fact that microplastics can be easily dispersed in the aquatic environment, the dredge sediments might contain significant amounts of microplastics [121].

More than 200 million m^3^ of dredge sediments have been collected annually from the bottom of almost 400 ports in the USA and mainly used for urban soil reconstruction and upland placement for agriculture [123]. In another study, the dredge sediment that was mainly composed of fine sand was collected from a riverbank in Dhaka, Bangladesh and used as filling material for land reclamation [124]. The total sediment amount that has been dredged annually in China is more than 5 billion m^3^ [125]. For instance, 136 and 116 million m^3^ of dredge sediments were collected from the riverbanks in Zhejiang Province, China for reconstruction facilities in 2016 and 2017, respectively [125,126]. Moreover, Ji et al. [121] mentioned that only 3 out of 10 million m^3^ of the collected dredge sediments from the riverbanks in Yueqing, China was transported and dumped into a landfill to serve as the cover soil in a coastal reclamation project. The remaining 7 million m^3^ of the dredge sediment was placed in storage piles to be used as agricultural fertilizers in farmlands. The microplastic content in the soil around the storage piles was found to be higher in the dry season due to the wind dispersion while the river surrounded by the storage piles contained higher microplastics in the wet season due to the surface run-off [121].

### 4.3. Soil Improvement with Synthetic Polymer-Based Fibers

Polymers have been used in fiber form for various geotechnical engineering applications in recent years, especially for ground improvement. Polymer-based fibers are typically added to the soil in order to enhance the strength parameters of the soil. These fibers serve as the coating agents that are able to fill the voids among the soil grains as well as act as reinforcements in soil fabric. Furthermore, these fibers can be mixed with other binding materials such as lime or cement in the soil for increasing the shear and compressive strengths of the soil [127]. In various studies, synthetic polymer-based fibers were added to clayey or sandy soils or soil-cement mixtures, and geotechnical laboratory tests such as unconfined compression, direct shear and triaxial compression tests were performed on these mixtures [128,129,130,131]. The test results indicated that fiber addition up to a limiting content led to an increase in both shear and compressive strengths of the soil, which indicates improved geotechnical properties for foundation design.

For example, Ayeldeen and Kitazume [132] added a synthetic polymer-based fiber (polypropylene) with a content of up to 1% by dry weight to a clayey soil that was mixed with cement. The cement content in the clay was 15% by dry weight. The fiber content of 0.5% caused the shear strength of the clay-cement mixture to increase by almost 240%. However, further increase in the fiber content resulted in a decrease in the shear strength. The synthetic polymer-based fibers can also be used as the secondary additives to increase the ductility of stabilized soils that are mainly used as barriers in landfills or subgrade soils beneath roadways [133]. Yılmaz and Sevencan [134] added polypropylene fiber to a soil mixed with fly ash and the fiber addition led to an increase in the unconfined compressive strength. In another study, polypropylene fibers and waste ashes were added to a sulfate-rich expansive soil. Due to the fiber addition, both the swell index and the shrinkage strain of the expansive soil increased [135]. Öncü and Bilsel [133] added a polymeric fiber with a content of 2% by dry weight to an expansive soil-sand mixture. According to the results of the laboratory tests, compressive and split tensile strength values of the soil mixture increased.

However, polymer-based fibers that are added to soils for reinforcement can easily be fragmented into microplastics [96,136]. In literature, polypropylene is reported to be one of the most abundant microplastic polymer types in both wastewater and soil environments [85,93,100,102,137]. Hence, one should remember that there is a considerable risk of microplastic contamination when soils are stabilized by the addition of synthetic polymer-based fibers for various civil/geotechnical engineering projects.

### 4.4. Geotechnical Applications with Expanded Polystyrene as a Lightweight Fill Material

#### 4.4.1. Polystyrene Based Microplastics in Soils

Polystyrene (PS) is the polymer type that is typically used for the manufacture of hard plastics such as food packages, food containers, beverage cups, plates and laboratory wares etc. [138,139]. However, once polystyrene-based plastic wastes are released to the environment, they can be broken into smaller pieces easily and carried to the atmosphere, water resources and terrestrial lands that are farther away from the location where the wastes are originally deposited or alternatively can penetrate into the soil vertically [100,140]. As mentioned in Section 2.3 of this paper, PS is also a very persistent polymer (Figure 3c) in soil environment.

A good example about the distribution of PS based microplastics can be seen in a case study from Belarus by Kukharchyk and Chernyuk [140], where an industrial plant deposited its plastic wastes into an area nearby a river. Soil and groundwater samples were collected from the floodplain of the river. High amounts of microplastics that were composed of polystyrene were detected in both the soil and the groundwater samples. The microplastic amount in the soil was measured as 1700 ptcl./kg while the groundwater samples contained 16,700 ptcl./kg microplastics [140]. Moreover, polystyrene particles smaller than 1 mm were found not only at the surface of the soil but also at a depth of 10–15 cm below the surface. Note that microplastic contamination at depths greater than 15 cm is unknown.

On the other hand, microplastics with a particle size of 0.001 mm (1 μm) that are formed by the disintegration of polystyrene-based plastic wastes, can also be considered as a serious threat for human beings if inhaled. Once the polystyrene based microplastic particles are inhaled or the foods that contain these particles are eaten, the polystyrene microplastics may destabilize red blood cells and eventually could lead to the disease named as hemolysis [138].

#### 4.4.2. Geotechnical Applications with Polystyrene Based Lightweight Fills

Expanded polystyrene is one of the most preferred lightweight fill materials that is used for geotechnical applications including embankment constructions for roads and bridges, slope stabilization and backfills of retaining walls [141,142,143,144]. In road and bridge constructions over soft soils, lightweight fill embankments with expanded polystyrene are used in order to reduce the bearing pressures on the soft soil [145,146]. Expanded polystyrene as a lightweight fill material is also preferred to be used to protect culverts and buried pipelines by decreasing the soil pressures acting on these structures [137,147].

In another study, prefabricated vertical expanded polystyrene drains were used for reducing the swelling capability of an expansive soil beneath the foundations of a couple of buildings in India [148]. After a while, expanded polystyrene drains stabilized the expansive soil by decreasing the rate of consolidation and settlements. Note that the mentioned drains in the study of Daigavane et al. [148] are not lightweight fills, but drainage systems (i.e., they are completely different geotechnical applications involving PS based materials).

Although the usage of the expanded polystyrene as a lightweight fill material in various geotechnical applications has several important benefits from engineering point of view, the possible propagation of the microplastics to the soil may lead to at least local soil and groundwater pollution in the long term.

It should be reminded that the geotechnical applications mentioned in Section 4 of this review paper (i.e., soil improvement with tire chips, forming engineering fills with dredged sediments, soil improvement with synthetic polymer-based fibers, polystyrene based lightweight fill applications) are all smart engineering solutions and beneficial for different civil engineering projects. Moreover, the level/weight of microplastic contamination in soils due to such applications is probably relatively small compared to the contamination levels caused by massive amounts of global plastic waste generated systematically. Therefore, the goal of authors is not to criticize or discourage those applications, but rather make the engineers be aware of the potential risks for local microplastic contamination. More importantly, authors suggest that future multidisciplinary research on microplastics should consider assessing and quantifying such potential risks.

## 5. Benefitting Geotechnical Engineering as a Mitigation Tool for Microplastics, Future Research and Concluding Remarks

In this review paper, microplastic contamination in soils is assessed from a geotechnical engineering perspective. It was emphasized that there is a delay of global awareness between different disciplines regarding the microplastic contamination in soils. A basic statistical analysis on the published literature for the last six years revealed that there are only two engineering disciplines (i.e., Chemical and Environmental Engineering) among the top 10 disciplines conducting research about microplastics and soils (Figure 2). Meanwhile, as civil/geotechnical engineers, authors of this paper could definitely conclude that the global awareness of the Civil Engineering community on microplastics is extremely limited, which is one of the main motivations for this review.

Today, it is well known that microplastic contamination in soils is a global problem that needs to be seriously considered. In the second part of this review, microplastic-soil relationship is addressed from degradation, existence and persistence aspects based on various studies in literature. Meanwhile, it was observed that the mentioned studies focused mostly on agricultural, environmental and health aspects, which are all very important. However, it would be quite beneficial to adopt the geotechnical engineering perspective in order to better understand the soil-microplastic interaction. For instance, influence of several relevant key parameters in geotechnical engineering, such as void ratio and hydraulic conductivity, are reminded for future multidisciplinary research on the migration and spatial distribution of microplastics in soils. It was also observed that the relevant literature on the microplastic existence in soils typically focused for the first 20 cm below the ground surface (with one exception for 30 cm). However, this depth range (0–30 cm) is very shallow from a geotechnical engineering perspective. Hence, more studies are needed to quantify the existence of microplastics at greater depths from the ground surface. In addition, consideration of the influence of different soil types used in engineering classification (e.g., clay, silt, sand, gravel and their mixtures) on microplastic migration and distribution in soils is needed in future multi-disciplinary research studies, the effects of which are currently unknown.

In the third part of this review, the geotechnical design of MSW landfills is addressed, including the compacted clay liners (CCL) and geosynthetic clay liners (GCL). Moreover, components of single and double-liner systems and their functions are explained (Figure 6). The limited amount of studies in literature clearly reveal that leachates from various landfills involve different concentrations of microplastics, which is highly expected considering the vast amount of plastic waste dumped in landfills globally. However, the number of studies on microplastic contamination in soils beneath or in the vicinity of landfills are even more limited (authors could find extremely few studies on the subject). Authors think that this subject is very important and deserves further research with case studies, because the lack of sufficient liner systems for abandoned waste dumps or defects/liner damages in landfills might also significantly contribute to the microplastic contamination in soils.

Furthermore, it would also be an interesting and critical research topic to investigate the performance of GCLs for filtering/holding the microplastics, which is currently unknown. It is possible that GCLs could act as a mitigation tool to reduce the microplastic contamination in soils. In fact, the authors of this paper have been working on a research project proposal on the topic. It is also ironic to remind that GCLs themselves also have polymer-based components (e.g., geotextiles), which could generate microplastics in the long term. However, authors think that their pros will be more than their cons both as a possible mitigation tool for microplastic contamination in soils and as an innovative engineering tool for various projects including landfills. Though, the mentioned hypothesis of authors should be evaluated in future studies. Moreover, the overall microplastic filtering/holding capacity of landfill liners working as a system (i.e., geotextile, drainage layer, geomembrane (with defect simulation), GCL working together as a system) should also be evaluated in future research.

In the last (fourth) part of this review, some geotechnical engineering applications as potential additional sources for microplastics in soils are expressed. These are soil improvement with tire chips, forming engineering fills with microplastic contaminated dredged sediments, soil improvement with synthetic polymer-based fibers, polystyrene based lightweight fill applications. Note that, there could be more geotechnical engineering applications which are not considered in this review. It is important to mention that those applications are all smart engineering solutions and beneficial for different civil engineering projects. Moreover, the weight of microplastic contamination in soils due to such applications is expected to be relatively very small compared to the weight of contamination levels caused by massive amounts of global plastic waste generated systematically. However, Civil/Geotechnical Engineers should be aware of the potential risks for additional microplastic contamination due to such applications. This could also be a novel multidisciplinary research topic, for which no studies are currently available in the literature.
